# Supplementary material for: Optimizing genomic sampling for demographic and epidemiological inference with Markov decision processes
Source: Genetics. 2025 Nov 11;232(1):iyaf244. doi: 10.1093/genetics/iyaf244 (PMC12774829; doi:10.1093/genetics/iyaf244)
Supplement: iyaf244_Supplementary_Data [file iyaf244_supplementary_data.pdf]

Supplemental Text: Optimizing genomic sampling for demographic and epidemiological inference with Markov decision processes

David A. Rasmussen[1,2], Madeline G. Bursell[2] and Frank Burkhart[2]

[1]Dept. of Entomology and Plant Pathology, North Carolina State University, Raleigh, NC, 27607, USA
[2]Bioinformatics Research Center, North Carolina State University, Raleigh, NC, 27607, USA

*Corresponding author: David A. Rasmussen (drasmus@ncsu.edu)

Abstract

Keywords:

Supplemental Text 1: Optimization using dynamic programming

To evaluate the expected value of a given policy π, we use an iterative policy evaluation algorithm (Sutton and Barto 2018). Starting from an initially arbitrary estimate of the value $V(s_t)$ for each state $s_t \in \mathcal{S}$ at each time $t = 1, ..., N$, each iteration of the algorithm updates the estimate of $V(s_t)$ using the expected value of successor states $V(s_{t+1})$ obtained from previous iterations. This procedure is iterated until the algorithm converges on a stable estimate of V.

Algorithm 1 Iterative Policy Evaluation

Input: The policy π to be evaluated and an accuracy threshold parameter $\theta > 0$

$V(s_t) \leftarrow$ RAND for each $s_t \in \mathcal{S}$
$V(s_{N+1}) \leftarrow 0$
while $\Delta > \theta$: **do**
  $\Delta \leftarrow 0$
  for *each* $s_t \in \mathcal{S}$: **do**
    $v \leftarrow V(s_t)$
    $V(s_t) \leftarrow \sum_a \pi(a|s_t) \sum_{s_{t+1}} p(s_{t+1}|s_t, a)\left[r(s_t, a) + V(s_{t+1})\right].$
    $\Delta \leftarrow \max(\Delta, |v - V(s_t)|)$
Output: V

To identify the optimal policy, we use a value iteration algorithm (Alagoz *et al.* 2010; Sutton and Barto 2018). This algorithm combines iteratively updating expected values with improvements to the policy. While the policy is not explicitly updated, the values are always updated with the expected value of the action that would maximize long-term expected value, such that the policy implicitly improves over time. This procedure is iterated until the algorithm converges on a stable estimate of V where the policy can no longer be improved. Given the optimized value function V, we can then find the optimal action a^* to take from any state s_t: $a^* = \arg\max_a [q_\pi(s_t, a)]$. Identifying a^* for all possible states therefore provides the optimal policy.

Algorithm 2 Value Iteration

Input: Accuracy threshold parameter $\theta > 0$

$V(s_t) \leftarrow$ RAND for each $s_t \in \mathcal{S}$
$V(s_{N+1}) \leftarrow 0$
while $\Delta > \theta$: **do**
  $\Delta \leftarrow 0$
  for *each* $s_t \in \mathcal{S}$: **do**
    $v \leftarrow V(s_t)$
    $V(s_t) \leftarrow \max_a \sum_{s_{t+1}} p(s_{t+1}|s_t, a)\left[r(s_t, a) + V(s_{t+1})\right].$
    $\Delta \leftarrow \max(\Delta, |v - V(s_t)|)$
for *each* $s_t \in \mathcal{S}$: **do**
  $\pi^*(s_t) \leftarrow \arg\max_a \sum_{s_{t+1}} p(s_{t+1}|s_t, a)\left[r(s_t, a) + V(s_{t+1})\right]$
Output: *The optimal policy* π^*

For MDPs with a finite number of states and actions, policy evaluation is guaranteed to converge to V as the number of iterations $k \to \infty$ (Sutton and Barto 2018). Value iteration is also guaranteed to converge to an optimal policy in a finite number of iterations (Sutton and Barto 2018). However, the optimal policy may not be unique. We therefore assess global convergence to π^* by starting the algorithm from several different initial values for $V(s_t)$.

Supplemental Text 2: Exponential growth coalescent MDP

In order to compute the expected value of a new sample under the exponential coalescent model, we track the probability density $p(\tau|z_{1:t})$ for the time at which the newly sampled individual coalesces with the other sampled lineages, conditional on the sample configuration $z_{1:t}$.

Lineages through time density

To obtain $p(\tau|z_{1:t})$, we first need to consider the lineage through time (LTT) density $q(l,\tau)$, which gives the probability that l other sampled lineages are present at time τ in the past.

In a single generation, the probability that a single pair of lineages coalesces is $1/N(\tau)$, such that the probability a given pair does not coalesce is $1-1/N(\tau)$. With k lineages present, there are $\binom{l}{2}$ pairs of lineages which could potentially coalesce. Assuming more than one coalescent event cannot occur per generation, the transition probabilities for how the number of lineages changes between generations are:

$$p(L_{\tau+1}=l|L_\tau=l)=1-\frac{\binom{l}{2}}{N(\tau)},$$
$$p(L_{\tau+1}=l-1|L_\tau=l)=\frac{\binom{l}{2}}{N(\tau)}.$$

We can iteratively compute $q(l,\tau)$ backward in time given these transition probabilities, or we can approximate these probabilities in continuous-time by solving the following system of differential equations:

$$\frac{dq(l,\tau)}{d\tau}=\frac{\binom{l+1}{2}}{N(\tau)}q(l+1,\tau)-\frac{\binom{l}{2}}{N(\tau)}q(l,\tau). \quad (1)$$

Sequential sampling through time can be accommodated by updating $q(l,\tau)$ at sampling events to reflect the addition of new lineages. If at time τ in the past z lineages are sampled, we update $q(l,\tau)$ as:

$$q(l+z,\tau)=q(l,\tau). \quad (2)$$

Numerically solving $q(l,\tau)$ through time shows that the computed lineage through time probabilities match the trajectories of lineages through time in Monte Carlo simulations of the coalescent process (Supplementary Figure 1A).

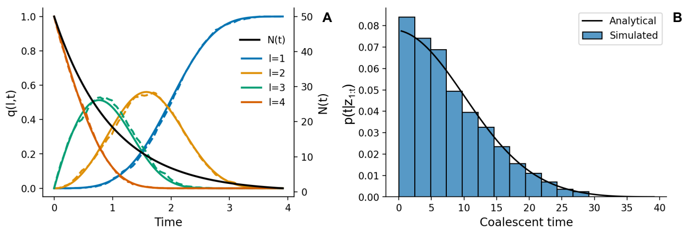

Supplementary Figure 1 Computing the LTT and coalescent time density for the exponential growth MDP. **A**: The probability density $q(l,\tau)$ for the number of lineage l through time (solid lines) compared with Monte Carlo simulations of the coalescent process (dashed lines) with 4 lineages sampled at present. **B**: The probability density $p(\tau|z_{1:t})$ for the time t_{coal} at which a sampled lineage coalesces with the rest of the sample compared against Monte Carlo simulations. Here, $p(\tau|z_{1:t})$ was computed using the LTT density $q(l,\tau)$ shown in B.

Coalescent time probability density

As originally shown in Slatkin and Hudson (1991), the probability that a pair of sampled lineages coalesce at time τ in the past in an exponentially growing population is:

$$p_{pair}(\tau)=\frac{1}{N(\tau)}\exp\left(-\int_0^\tau \frac{1}{N(\tau')}d\tau'\right). \quad (3)$$

Modifying this density to take into account any possible number of sampled lineages l through time, the probability density $p(\tau|z_{1:t})$ for the time at which a sampled lineage coalesces with the rest of the sample is:

$$p(\tau|z_{1:t})=\sum_{l=1}^{l=L_{max}}\frac{lq(l,\tau)}{N(\tau)}\exp\left(-\int_0^\tau\sum_{l=1}^{l=L_{max}}\frac{lq(l,\tau)}{N(\tau')}d\tau'\right), \quad (4)$$

where $L_{max}=\sum_{t=1}^{t=N}z_t$, the maximum possible number of samples in the tree.

Supplementary Figure 1B shows the probability density for the time at which a newly sampled lineage coalesces with the rest of the sample for the case where four other lineages are sampled at present. The analytical density given by Eq (4) closely follows the observed distribution of coalescent times from Monte Carlo simulations.

Supplemental Text 3: Transmission tree distance MDP

In order to compute the expected value of a given sampling action in the transmission tree MDP, we track two auxiliary probability densities backwards through time. The first tracks the number of individuals or lineages ancestral to the sample at each generation. We refer to this as the lineages through time (LTT) density. The second tracks the transmission distance between a representative ancestor and its nearest sampled descendant. We refer to this as the ancestor-to-sample distance density.

Note that, as a shorthand below, we refer to any individual that has a sampled descendant as a sampled ancestor, regardless of whether that individual is directly sampled or not.

Lineages through time density

Let $q(l,t)$ be the probability that l sampled ancestors are present in the transmission tree at time t, conditional on the number of samples taken at each generation $z_{1:t}$. Going backwards in time, the number of sampled ancestors can change due to coalescent events where two or more sampled ancestors share the same parent in the previous generation.

Given $L_t=k$ sampled ancestors at time t, the probability that there are $L_{t+1}=l$ sampled ancestors one generation in the past is:

$$p(L_{t+1}=l|L_t=k)=g(l|k)=\frac{s_2(k,l)\prod_{x=0}^{x=j+1}N-x}{N^k},$$

where $s_2(k,l)$ is the Stirling number of the second kind and N is the population size (Watterson 1975; Wakeley 2009). Note that these transition densities account for the possibility of multiple lineages coalescing in a single generation, which may be likely when the number of sampled ancestors is large relative to N.

Given the transition densities $g(l|k)$, we can update $q(l,t)$ at each generation going into the past using the following recursion:

$$q(l,t+1)=\sum_{k\neq l}\Big(g(l|k)q(k,t)-g(k|l)q(l,t)\Big). \quad (5)$$

The number of sampled ancestors can also change due to sampling events at each generation. However, not all samples will necessarily increase the number of individuals ancestral to sample because a newly sampled individual may already be a sampled ancestor. In other words, the sampled individual may already have a sampled descendant in the sample. Assuming

that there are l sampled ancestors prior to the sampling event, the probability that a randomly sampled individual is already a sampled ancestor is $p_{anc} = l/N$. Given z_t new samples, the probability that k of these individuals are newly sampled ancestors not already in the sample follows a binomial distribution:

$$u(k|z_t) = \binom{z_t}{k}(1 - p_{anc})^k p_{anc}^{z_t - k}. \tag{6}$$

We can therefore update $q(l,t)$ for all possible values of l at sampling events by considering how many of the z_t sampled individuals are newly sampled ancestors:

$$q(l,t) = \sum_{k=0}^{k=z_t} q(l-k,t)u(k|z_t). \tag{7}$$

Solving the recursion for $q(l,t)$ backwards through time shows that the lineage through time probabilities match the equivalent probabilities computed from simulated transmission trees (Supplementary Figure 2A).

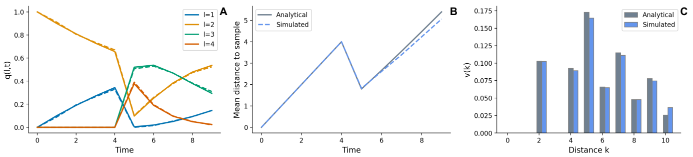

Supplementary Figure 2 Probability densities used to compute expected rewards under the transmission tree MDP. **A**: The probability density $q(l,t)$ for the number of lineages through time (solid lines) compared with Monte Carlo simulations of transmission trees (dashed lines). **B**: The mean ancestor-to-sample distance computed based on the probability density $m(k,t)$ (solid line) compared with Monte Carlo simulations (dashed line). **C**: The probability density $v(k|\mathcal{Z}_{1:t})$ that a sample has its nearest sampled neighbor at transmission distance k. In all cases, $N(0) = 10$ with with 2 lineages sampled at present ($t = 0$) and 2 lineages sampled at $t = 4$.

Ancestor-to-sample distance density

We also track the transmission distance between a representative sampled ancestor and its nearest directly sampled descendant. Conditional upon an individual being a sampled ancestor, let $m(k,t)$ be the probability that the transmission distance between this individual and its nearest sampled descendant is k. For a newly sampled individual $m(k,t) = 0$. Without further sampling, the transmission distance between our representative ancestor and its sampled descendant increases by one each generation going into the past, such that $m(k+1,t+1) = m(k,t)$ for all k.

However, if we are sampling sequentially though time, the distance of our representative ancestor to its nearest sampled descendant will reset to zero if it is sampled again at an earlier generation (i.e. if it is resampled). We therefore need to compute the probability that our representative ancestor is among the individuals sampled at a sampling event. This is complicated by the fact that we do not necessarily know the total number of sampled ancestors in the transmission tree before the sampling event (but only $q(l,t)$) nor the number of new sampled ancestors the sampling event will add to the sample (because we may resample any already sampled ancestor). Nevertheless, we can compute the overall probability that our representative ancestor is among the z_t newly sampled individuals by summing over all possible numbers of total ancestors l and newly sampled ancestors k:

$$p_{sampled} = \sum_{l=0}^{l=L_{max}} \sum_{k=0}^{k=z_t} q(l,t)u(k|z_t)\frac{z_t}{l+k}. \tag{8}$$

We then update $m(k,t)$ after the sampling event by setting $m(0,t) = p_{sampled}$ and $m(k,t) = (1 - p_{sampled})m(k,t)$ for $k > 0$.

The distance of our representative ancestor to its nearest sampled descendant may also change if it coalesces with another lineage that has a closer sampled ancestor. To account for this possibility, we first compute that probability that our representative ancestor coalesces with any of the other l sampled ancestors in a given generation:

$$p_{coal} = \sum_{l=1}^{l=L_{max}} q(l,t)\frac{l-1}{N}. \tag{9}$$

Conditional upon our representative ancestor coalescing with another sampled lineage, let $m(l,t)$ be the distance of the other lineage to its nearest sampled descendant. Assuming all lineages in the sample are exchangeable, $m(l,t) = m(k,t)$. The probability that the other lineage is closer to its nearest sampled descendant than our representative ancestor is therefore $\sum_{l=0}^{l=k-1} m(l,t)$. Likewise, the probability that the other lineage is further away from its nearest sampled descendant is $\sum_{l=k+1}^{l=\infty} m(l,t)$. We can then combine these probabilities to update $m(k,t)$ at each generation going into the past using the following recursion:

$$m(k,t+1) = m(k,t) + \frac{m(k,t)p_{coal}}{2}\left(\sum_{l=k+1}^{l=\infty} m(l,t) - \sum_{l=0}^{l=k-1} m(l,t)\right). \tag{10}$$

Note that we divide by two here since the probability that our representative ancestral lineage is the lineage with the lower of the two distances is $1/2$.

Solving the recursion for $m(k,t)$ backwards through time gives mean ancestor-to-sample distances. The resulting distance strongly agree with the mean distances computed from simulated transmission trees (Supplementary Figure 2B).

Transmission distance probabilities

Given $q(l,t)$ and $m(k,t)$, we can combine these two probability densities to compute the probability $v(k|\mathcal{Z}_{1:t})$ that a newly sampled individual had a nearest sampled neighbor in $\mathcal{Z}_{1:t}$ at any distance k.

To compute $v(k|\mathcal{Z}_{1:t})$, we first consider the probability $w(k)$ that a sampled individual i has a sampled neighbor j at some transmission distance $d(i,j) = k$. In order for $d(i,j) = k$, i and j must share a common ancestor h at some t_{coal} where the transmission distances $d(i,h)$ and $d(j,h)$ sum to k. We can therefore think about k as being the sum of two random variables, $x = d(i,h)$ and $y = d(j,h)$), determined by the unobserved topology of the transmission tree.

If individual i is sampled at time t_{sample}, it must coalesce with another sampled lineage at time $t_{coal} = t_{sample} + x$ in order for $x = d(i,h)$. The probability that lineage i coalesces with another sampled lineage at t_{coal} is:

$$c(t_{coal}) = \sum_l q(l,t_{coal})\frac{l}{N}. \tag{11}$$

Given a coalescent event at this time, the probability that the other child lineage has a sampled descendant at transmission distance $y = d(j, h)$ is provided by $m(y, t_{coal})$.

To compute $w(k)$, we can then sum the probability of all coalescent events for which $x + y = d(i, h) + d(j, h) = k$:

$$w(k) = \sum_{x=0}^{x=k} c(t_{sample} + x) m(k - x, t). \quad (12)$$

However, $w(k)$ simply represents the probability that a sampled individual has a sampled neighbor at transmission distance k. To compute the probability that a sampled individual's *nearest* sampled neighbor is at transmission distance k, we need to to consider the (cumulative) probability that the sampled individual does not have a sampled neighbor at a distance less than k, but does have a sampled neighbor at distance k. We therefore arrive at our desired probability density:

$$v(k|\mathcal{Z}_{1:t}) = w(k) \prod_{l=1}^{l=k-1} [1 - w(l)]. \quad (13)$$

The probability density $v(k|\mathcal{Z}_{1:t})$ strongly agrees with probabilities of a randomly sampled individual having a nearest sampled neighbor at distance k in simulated transmission trees (Supplementary Figure 2C).

Supplemental Text 4: Structured coalescent MDP

In order to compute the expected value of a new sample under the structured coalescent, we track the joint probability density $p(\tau, u, v|z_{1:t})$ for the time τ in the past at which the sampled individual coalesces with the rest of the sample along with the state u of the parent lineage and state v of the child (sampled) lineage at the coalescent event. As with the exponential coalescent MDP, computing the coalescent density $p(\tau, u, v|z_{1:t})$ requires tracking the number of lineages through time (LTT) density backwards through time, but now for the number of lineages in each population.

Lineages through time density

For the structured coalescent model with two populations, let $q(k, l, \tau)$ be the probability that k sampled lineages are in population 1 and l sampled lineages are in population 2 at time τ in the past. To track the number of lineages through time, we distinguish between three different types of coalescent events, as each event type causes the number of lineages to change differently.

- **Type 1:** Coalescent events between lineages in the same population. These events will cause the number of lineages to decrease by one in that population.
- **Type 2:** Coalescent events between lineages in different populations. In this case, one of the two child lineages will be in the same population as the parent lineage, while the other child will be in the other population. The number of lineages will decrease by one in the non-parental population.
- **Type 3:** Unobserved coalescent events arising from one of the two child lineages not being sampled. If the sampled child lineage is in a different state from its parent, the sampled lineage moving between populations at an unobserved coalescent event. The number of lineages in the child population will go down by one and go up by one in the parental population.

Let $L_\tau = (k, l)$ denote the configuration of lineages at time τ, where k is the number of sampled lineages in population 1 and l is the number in population 2. Given L_τ, we consider the probability of transitioning to $L_{\tau+1}$ at time $\tau + 1$ one generation in the past. Assuming more than one event cannot occur per unit of time, the transition probability densities are:

$$
\begin{aligned}
p\Big(L_{\tau+1} = (k-1, l)|L_\tau = (k, l)\Big) &= \binom{k}{2} \frac{1 - m_{2,1}}{N_1(\tau)} && \text{(Type 1)}\\
p\Big(L_{\tau+1} = (k, l-1)|L_\tau = (k, l)\Big) &= \binom{l}{2} \frac{1 - m_{1,2}}{N_2(\tau)} && \text{(Type 1)}\\
p\Big(L_{\tau+1} = (k-1, l)|L_\tau = (k, l)\Big) &= kl \frac{m_{2,1}}{N_2(\tau)} && \text{(Type 2)}\\
p\Big(L_{\tau+1} = (k, l-1)|L_\tau = (k, l)\Big) &= kl \frac{m_{1,2}}{N_1(\tau)} && \text{(Type 2)}\\
p\Big(L_{\tau+1} = (k+1, l-1)|L_\tau = (k, l)\Big) &= l \frac{m_{1,2}}{N_1(\tau)} && \text{(Type 3)}\\
p\Big(L_{\tau+1} = (k-1, l+1)|L_\tau = (k, l)\Big) &= k \frac{m_{2,1}}{N_2(\tau)} && \text{(Type 3)} \quad (14)
\end{aligned}
$$

Based on these transition densities, we obtain the the following system of differential equations to track how $q(k, l, \tau)$ evolves backwards in continuous time:

$$
\begin{aligned}
\frac{dq(k, l, \tau)}{dt} = &\binom{k+1}{2} \frac{1 - m_{2,1}}{N_1(\tau)} q(k+1, l, \tau) - \binom{k}{2} \frac{1 - m_{2,1}}{N_1(\tau)} q(k, l, \tau)\\
&+ \binom{l+1}{2} \frac{1 - m_{1,2}}{N_2(\tau)} q(k, l+1, \tau) - \binom{l}{2} \frac{1 - m_{1,2}}{N_2(\tau)} q(k, l, \tau)\\
&+ (k+1) l \frac{m_{2,1}}{N_2(\tau)} q(k+1, l, \tau) - kl \frac{m_{2,1}}{N_2(\tau)} q(k, l, \tau)\\
&+ k(l+1) \frac{m_{1,2}}{N_1(\tau)} q(k, l+1, \tau) - kl \frac{m_{1,2}}{N_1(t)} q(k, l, \tau)\\
&+ (l+1) \frac{m_{1,2}}{N_1(\tau)} q(k-1, l+1, \tau) - l \frac{m_{1,2}}{N_1(\tau)} q(k, l, \tau)\\
&+ (k+1) \frac{m_{2,1}}{N_2(\tau)} q(k+1, l-1, \tau) - k \frac{m_{2,1}}{N_2(\tau)} q(k, l, \tau). \quad (15)
\end{aligned}
$$

Numerically solving $q(k, l, \tau)$ through time shows that the expected trajectory of lineages in each population through time matches the trajectories in Monte Carlo simulations of the structured coalescent process (Supplementary Figure 3A).

Structured coalescent density

The probability density $p(\tau, u, v|z_{1:t})$ can be computed iteratively at each time step in a three step process.

First, we compute the expected number of lineages in each population $A_1(\tau)$ and $A_2(\tau)$ at time τ. These expected values can be computed from $q(k, l, \tau)$ by marginalizing over all possible configurations of lineages between the two populations:

$$
\begin{aligned}
A_1(\tau) &= \sum_{k=0}^{k=L_{max}} k \sum_{l=0}^{l=L_{max}} q(k, l, \tau),\\
A_2(\tau) &= \sum_{l=0}^{l=L_{max}} l \sum_{k=0}^{k=L_{max}} q(k, l, \tau). \quad (16)
\end{aligned}
$$

Second, we track the probabilities $s_1(\tau)$ and $s_2(\tau)$ that the lineage ancestral to the newly sampled individual resides in population 1 or 2, respectively. The probability that the sampled

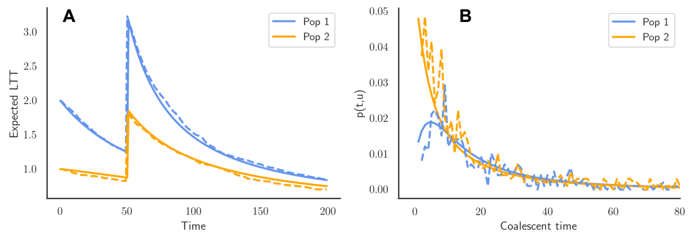

Supplementary Figure 3 The LTT and coalescent densities for the structured coalescent MDP. **A**: The expected number of lineages in each population through time computed from the LTT density $q(k, l, \tau)$ (solid lines) compared with Monte Carlo simulations of the coalescent process (dashed lines). In this case, two lineages are sampled from population 1 and one lineage is sampled from population 2, both at present and at time $\tau = 50$ in the past. **B**: The probability density $p(\tau, u|z_{1:t})$ for the time t_{coal} at which a lineage coalesces with a parent lineage in population $u = 1$ or population $u = 2$. The coalescent density computed using Eq 19 (solid lines) is compared against Monte Carlo simulations of the coalescent process (dashed lines). Here, two lineage are assumed to have already been sampled in each population at present ($t = 0$), and $p(\tau, u|z_{1:t})$ is given for a fifth lineage sampled in population 2.

lineage resides in each population can then be tracked using the following coupled pair of differential equations:

$$\begin{aligned} \frac{ds_1(\tau)}{d\tau} &= s_2(\tau)\frac{m_{1,2}}{N_1(\tau)} - s_1(\tau)\frac{m_{2,1}}{N_1(\tau)}, \\ \frac{ds_2(\tau)}{d\tau} &= s_1(\tau)\frac{m_{2,1}}{N_2(\tau)} - s_2(\tau)\frac{m_{1,2}}{N_2(\tau)}. \end{aligned} \quad (17)$$

Note that here we only consider the probability of the lineages moving between states due to unobserved (Type 3) coalescent events since Type 1 and 2 events would be observed in the phylogeny and we are only interested in what population the sampled lineage resides in before it coalesces with another sampled lineage.

Third, given $A(\tau)$ and $s(\tau)$, we compute the rate $\lambda_{u,v}(\tau)$ at which the sampled lineage coalesces at an event where the parent lineage is in state u and the child (sampled) lineage is in state v.

$$\lambda_{u,v}(\tau) = \begin{cases} s_u(\tau)A_u(\tau)\frac{1-\sum_y m_{y,u}}{N_u(\tau)}, & \text{if } u = v \\ s_v(t)A_u(t)\frac{m_{u,v}}{N_u(\tau)}, & \text{if } u \neq v. \end{cases} \quad (18)$$

The coalescent density $p(\tau, u, v|z_{1:t})$ is then obtained by considering the probability that no coalescent event occurs prior to time τ along with the probability that the sampled lineage coalesces with a parent in lineage in state u while in state v at time τ:

$$p(\tau, u, v|z_{1:t}) = \lambda_{u,v}(\tau)\exp\left(-\int_0^{\tau}\sum_{u\in\{1,2\}}\sum_{v\in\{1,2\}}\lambda_{u,v}(\tau')d\tau'\right). \quad (19)$$

Supplementary Figure 3B shows the probability density for the time at which a newly sampled lineage coalesces with a parent lineage in each population for the case where four other lineages (two in each population) are sampled at present. The density given by Eq (19) closely follows the observed distribution of coalescent times in each population observed in Monte Carlo simulations of the structured coalescent.

Literature cited

Alagoz O, Hsu H, Schaefer AJ, Roberts MS. 2010. Markov decision processes: a tool for sequential decision making under uncertainty. Medical Decision Making. 30:474–483.

Slatkin M, Hudson RR. 1991. Pairwise comparisons of mitochondrial dna sequences in stable and exponentially growing populations. Genetics. 129:555–562.

Sutton RS, Barto AG. 2018. *Reinforcement learning: An introduction*. MIT press.

Wakeley JH. 2009. Coalescent theory: an introduction. Roberts and Company. .

Watterson G. 1975. On the number of segregating sites in genetical models without recombination. Theoretical Population Biology. 7:256–276.
